# The Hospital Annual
*Burdett's Hospital Annual and Year Book of Philanthropy, 1891—1892. Containing a Review of the Position and Requirements of the Voluntary Charities, and an exhaustive Record of Hospital Work for the year. Edited by Henry C. Burdett, author of "Hospitals and Asylums of the World" "Hospitals and the State," &c. London: The Hospital (Limited), 140, Strand, W.C.


**Published:** 1891-10-24

**Authors:** 


### The Hospital Annual.*

The Hospital Annual has made for itself a definite position in the literature of philanthropy. It is the recognised book of reference for all manner of statistics connected with our charities; and while it must for this reason contain every year a large quantity of matter very similar to that which it held during the previous twelvemonth, the editor manages to secure for each new issue an amount of information for the introductory chapters which is as varied as it is valuable. There still remain certain defects in the work, but for these we cannot hold the editor responsible. We note with regret the occasional recurrence of the asterisk which shows that the secretary of an institution has failed to respond to the request made for information as to the size and work of the charity with which he is connected. In the case of those institutions which borrow the name of hospital to cover the cupidity of one unscrupulous man, we can understand such reticence; but we regret that in any other case the officials of a hospital, nursing institution, or dispensary should fail to appreciate the value of having the essential facts of their institution placed in what has become the recognised record of such matters. Also, as the "Hospital Annual" is referred to both by those engaged in the work of nursing and the geneial public, it is to be regretted that the heads of nursing institutions do not state both the amount they pay their nurses and the sum they charge for a nurse's attendance. As the fact that this "Annual" is referred to by all classes of the community is grasped by officials, there is little doubt that these defects will be remedied.

We may safely say that these are the only flaws in an interesting and valuable work. This year's issue contains a list of colonial hospitals, which cannot fail to be interesting to all who have friends in Greater Britain. As yet it is incomplete, but doubtless every year will see this department of the "Annual" enlarged and improved. Another new feature is that included in the section entitled the "Handbook of Philanthropy," which gives a list of institutions for the blind, crippled, imbeciles, inebriates, &c., which cannot fail to be useful. In fact we may venture to say that this part of the "Annual" alone gives it a right to be placed on the desk of every clergyman, doctor, or anyone else who is likely to be consulted as to the disposal of the diseased, afflicted, or degraded. It condenses in a lucid form an amount of information which is very often necessary and too frequently unattainable to the philanthropist.

With regard to the introductory chapters, the only thing we would deprecate is the attention given to the wire-pullers who tried with so much malevolence to influence the Lords' Committee of Inquiry. Since they have failed in their attempt to discredit honest workers and noble charities, it is better to leave them to the oblivion they deserve. But no one can read these chapters without perceiving the accurate knowledge, sound thought, and conscientious honesty that inspire them. There is no truckling to the fancies of officials who may want, not altogether selfishly, to bring money to the treasuries of the institutions to which they belong. Worthy of special note are those remarks on the needless increase of charities which is too common in our day: "There can be little doubt that a well-regulated city should establish some system of control whereby the multiplication of medical charities beyond the needs of the population could be prevented. It is folly to permit the present inchoate system to continue, whereby any man or woman can set up an establishment, and dub it a public medical charity for any class of disease, whether it is wanted or not. The managers of public institutions ought to take steps to prevent their abuse by people who can well afford to pay for their treatment when ill. In America, as in Sweden and Norway, every applicant is compelled to pay, unless proof is afforded that his claim to free medical relief is based upon genuine necessity."

It is a pity that the existence of a medical school in a town so often tends to the abuse of medical charities; but when they have pupils to teach, the main object of physicians and surgeons seems to be to collect a large number of patients for clinical instruction, and it appears to be nobody's business to ask if all these patients are really so circumstanced as to require free treatment. So great is this abuse in the out-patient department especially, that the compiler of the "Annual" goes so far as to say that in many cases it would be better if this department were wholly closed for a time. At present some towns have absolutely too many medical charities, and the result is a very considerable demoralisation of the population, who take all their ailments to the nearest hospital instead of calling in a doctor. Nor are these the towns where, owing to the nature of the prevailing industries, such a result might be regarded as natural. The busy town of Preston finds it necessary to provide only one bed, one hospital bed, for each thousand of the population, while Dublin, which is not specially an industrial centre, has no less than 6 39. The result is that while in Preston 11·7 in the thousand of population receive hospital treatment as in-patients, the proportion in Dublin is 45 9 per thousand. The out-patient departments are, as has been stated, even worse. In Dublin, Edinburgh, and Liverpool, nearly one in every two of population receives free medical relief. Can such a state of things be regarded as other than scandalous?

The endless subject of hospital finance is admirably treated, and though we are far from thinking or suggesting that cheapness is everything in the administration of charities or anything else, we feel sure that the officials of several London hospitals may find cause for self-searching and study in the combination of efficiency and economy which is shown by many provincial and Scotch institutions. Much might be done if any system of uniformity in book-keeping were adopted, and as an aid to this, we note with great satisfaction the sample pages from account books which are given. They show a system which is admirably clear, detailed without being diffuse, and revealing at a glance on what departments of the hospital the money is chiefly spent. We specially commend these pages to hospital secretaries, who are doubtless often at a loss to know under what heads to classify the different items of expense in an institution so large and complicated as a hospital. The index of classification compiled by Mr. Michelli is a valuable addition to these pages.

The chapter on nursing ought to be read by all who think of adopting that profession. They give the aspirant every information as to how to become a nurse, what to do when she is one, and what remuneration she may expect at every stage of her career. The information given about the National Pension Fund should make it impossible for even the dullest to cherish longer the erroneous ideas so industriously spread about this noble scheme; and the subject of registration for nurses and midwives respectively is plainly dealt with. In fact, this chapter is a model of clearness, compactness, and completeness. The whole volume is indeed as practical as it is interesting, and even more than its predecessors, the "Hospital Annual" of 1891-92 should be of the widest utility.

* Burdett's Hospital Annual and Year Book of Philanthropy, 1891—1892. Containing a Review of the Position and Requirements of the Voluntary Charities, and an exhaustive Record of Hospital Work for the year. Edited by Henry C. Burdett, author of "Hospitals and Asylums of the World" "Hospitals and the State," &c. London: The Hospital (Limited), 140, Strand, W.C.

